# A novel nano-particle strengthened titanium alloy with exceptional specific strength

**DOI:** 10.1038/s41598-019-48139-8

**Published:** 2019-08-13

**Authors:** Aniket K. Dutt, Bharat Gwalani, Vedavyas Tungala, Matthew Carl, Rajiv S. Mishra, Sesh A. Tamirisakandala, Marcus L. Young, Kyu C. Cho, Raymond E. Brennan

**Affiliations:** 1 Center for Friction Stir Processing, Department of Materials Science and Engineering, University of North Texas, Denton, TX 76207 USA; 2 Arconic, 1000 Warren Avenue, Niles, OH 44446 USA; 3 Weapons and Materials Research Directorate, U.S. Army Research Laboratory, Aberdeen Proving Grounds, Aberdeen, MD 21005 USA

**Keywords:** Mechanical properties, Metals and alloys

## Abstract

Various ecological and economical concerns have spurred mankind’s quest for materials that can provide enhanced weight savings and improved fuel efficiency. As part of this pursuit, we have microstructurally tailored an exceptionally high-strength titanium alloy, Ti-6Al-2Sn-4Zr-6Mo (Ti6246) through friction stir processing (FSP). FSP has altered the as-received bimodal microstructure into a unique modulated microstructure comprised of fine acicular α″-laths with nano precipitates within the laths. The sequence of phase transformations responsible for the modulated microstructure and consequently for the strength is discussed with the help of scanning electron microscopy, transmission electron microscopy, and synchrotron X-ray diffraction studies. The specific strength attained in one of the conditions is close to 450 MPa m^3^/mg, which is about 22% to 85% greater than any commercially available metallic material. Therefore, our novel nano particle strengthened Ti alloy is a potential replacement for many structural alloys, enabling significant weight reduction opportunities.

## Introduction

Over the past 70 years of titanium alloy research, improved and expanded utilization of these alloys have occured, largely due to their versatile properties, which include high specific strength, high temperature strength, and unparalleled corrosion resistance^1^. More recently, the various options for processing β-titanium alloys have led to their increased use in aerospace, automotive and biomedical industries^2^. For example, Boeing 787 passenger aircraft recently introduced a high strength alloy Ti-5Al-5Mo-5V-3Cr that replaced high-strength steels such as 300 M and 4340 for some of its weight-critical and performance driven applications^3^. High-performance sports cars and superbikes use β-titanium alloys for the majority of their engine components, mostly targeting enhanced weight savings^4,5^. Lower elastic modulus coupled with superior bio-corrosion resistance in the human body make these classes of alloys attractive choices for biomedical applications such as hip and joint replacements^6^.

With the ever-growing drive towards the aforementioned applications, improvement in mechanical properties becomes indispensable, and majority of applications require that the alloys be used in the processed condition. Ti-6Al-2Sn-4Zr-6Mo (Ti6246) is a high-strength, near β-titanium alloy^7^ that is used primarily in the intermediate temperature of gas turbine aero engines, as well as in some hot sections of race car engines^8^. However, joining/processing of this alloy is reported to be difficult because of the amount of β-stabilizing content it possesses^9^. In addition, literature on microstructural behavior after joining has been very limited. Thus, it is technologically important to investigate the feasibility of a solid-state processing technique such as friction stir processing (FSP), on this alloy. Furthermore, this investigation can expand the usage of this alloy, which possesses a strength level on par with ultra-high strength steels. In this study, we report a strength of approximately 2 GPa for Ti6246 processed under FSP. The strength value is approximately 60% higher than that of all other reported commercially-available titanium alloys^10^.

This study provides fundamental insight into the type of microstructure that is responsible for the high degree of strength obtained. In addition, a unique FSP path is proposed for all commercially available titanium alloys, that could give rise to significant improvements in strength, potentially serving many structural applications, and enabling a new domain for alloy design and development.

## Tensile Properties of Ti6246

**Figure 1:**
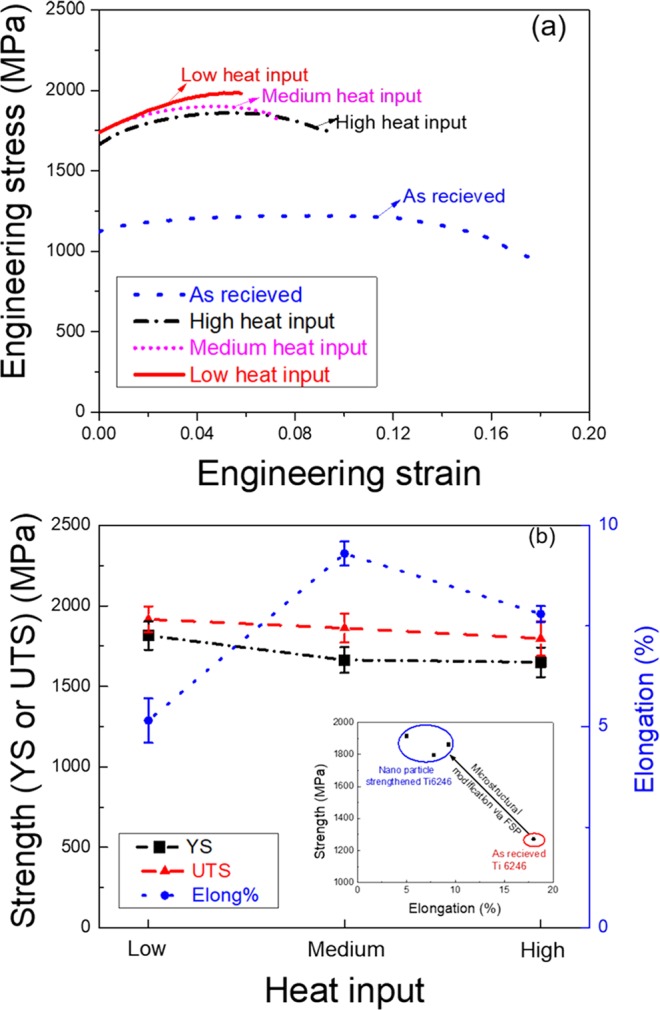
(**a**) Engineering stress-strain plots of Ti6246, as per indicated conditions; (**b**) Variation of mechanical properties as a function of heat input.

Ever since its inception, friction stir welding (FSW) has seen significant industrial usage, especially for the successful joining of many Al^11–16^, Mg^17,18^, and Cu^19^ alloys. However, the joining of titanium alloys is limited due to the availability of tool materials that can withstand the temperatures and loads involved in the process^20,21^. Bead-on-plate FSP runs were successfully carried out on Ti6246 (refer to “Methods” section for a detailed description of the process). Engineering stress – strain plots for the as-received (AR) and processed material at low, medium, and high heat inputs are shown in Fig. 1(a) (refer to the “Methods” section for heat input description). The variation of tensile properties under three different heat inputs shows that with increased heat input, the yield strength (YS) and the ultimate tensile strength (UTS) values decreased from 1816 ±8  MPa to 1650 ±9  MPa and from 1916 ±8  MPa to 1797 ±10  MPa, respectively; whereas elongation values increased from 5 to 10% (Fig. 1(b)). The most remarkable observation is UTS of 1996 MPa, with a 5% elongation obtained at low heat input. This strength level is approximately 65% higher than the base material (1272 MPa). The following sections describe phenomenal strengthening by correlating with microstructural features at various length scales.

## Microstructural Characterization

**Figure 2:**
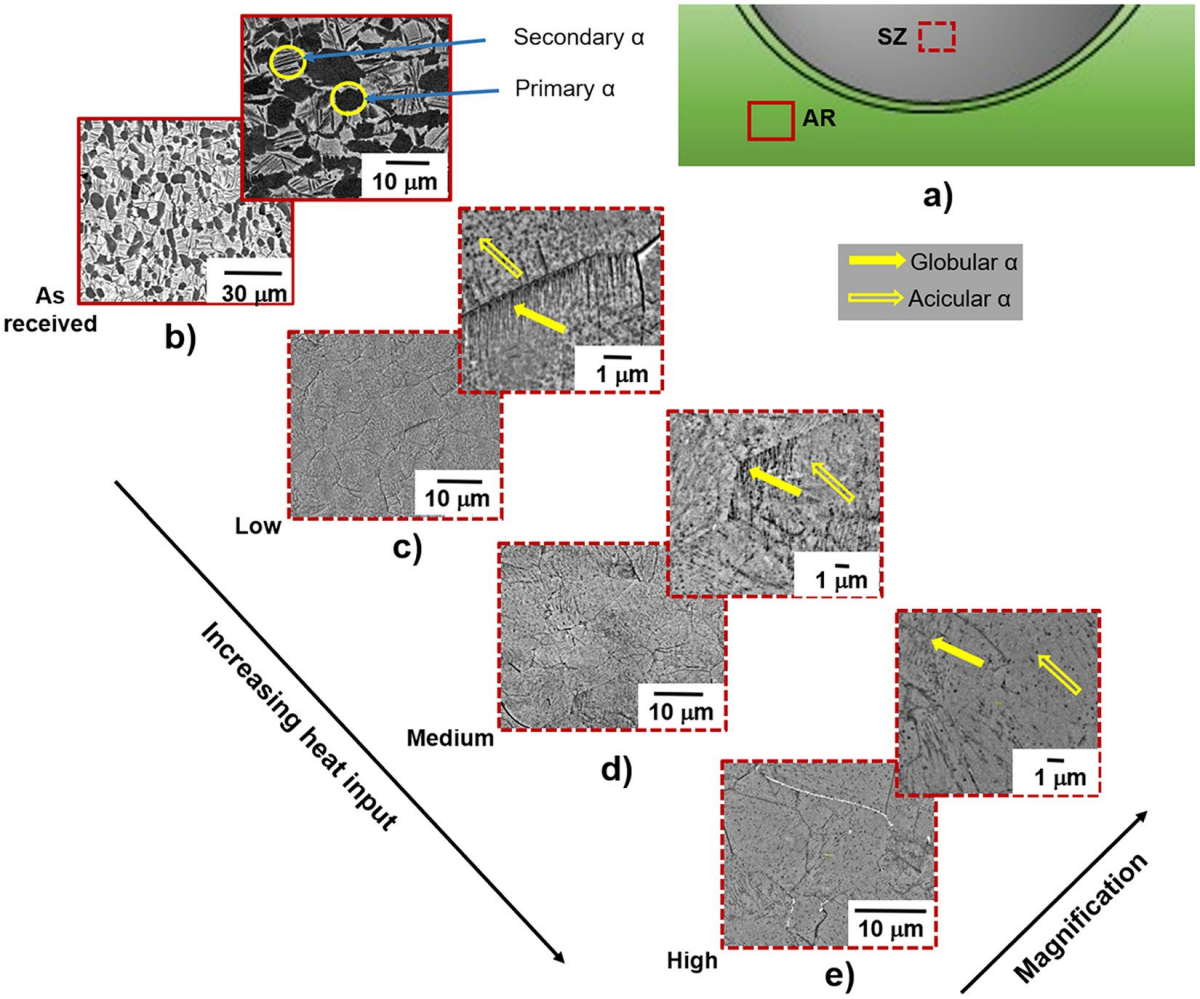
(**a**) Schematic of the transverse cross section with a dotted square in the shaded area indicating that the processed samples were taken approximately 1 mm from the top surface, while the thick square at the bottom indicates the approximate location of AR microstructure; (**b**) BSE image of AR material at low and high magnifications, in which arrows highlight primary α and secondary α phase morphologies; (**c**–**e**) showcase low and high magnification BSE images of the samples processed at low, medium, and high heat inputs, in which the closed arrows indicate fine globular-type α and the open arrows indicate acicular α-morphology.

To investigate the exceptional property combinations after FSP, scanning electron microscopy (SEM) was initially performed on the AR material, as well as the specimens extracted from the transverse cross-section of the processed material, as shown in the schematic of Fig. 2(a). The AR Ti6246 microstructure was bimodal (duplex), comprised of equiaxed primary α (α_p_)grains (volume fraction approximately 40%) at prior β-grain triplets and secondary α (lamellae of α) in a transformed β-matrix (Fig. 2(b)). The grain size of primary α and secondary α, 7.16 ±2.86 µm and 3.45 ±1.69 µm, respectively, were measured using image J software. The mechanical properties of this alloy depend primarily on the colony size of secondary-α lamellae^22^. Figure 2(c–e) show the backscattered scanning electron (BSE) images obtained from the stir zone (SZ) of transverse cross section at three different heat inputs. The high strain rate deformation from FSP created complex microstructure comprised of various morphologies of precipitates at different length scales in a prior β-grain matrix. The contrast difference between the phases was not quite discernible, likely due to the enhanced diffusion associated with FSP.

**Figure 3:**
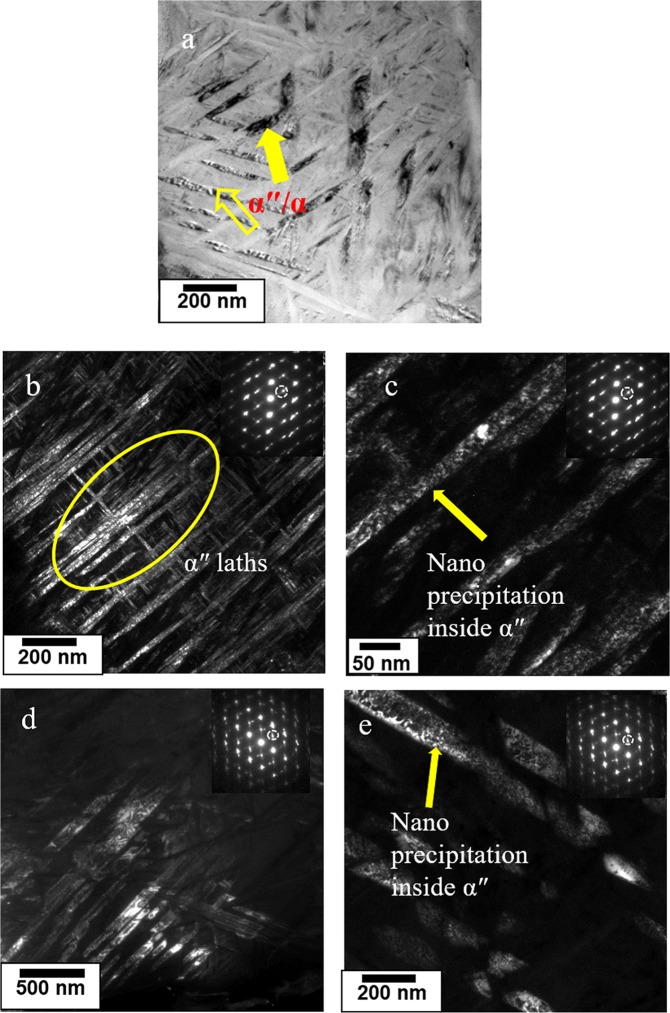
(**a**) Bright field TEM image of the sample processed at low heat input showing fine α and α″ laths; (**b**) Dark field TEM image taken from <110> zone axis of the sample processed at low heat input showing a modulated microstructure; (**c**) High magnification dark field image showing nano-scale β precipitation within the α″-lath; (**d**) Dark field TEM image taken from <110> zone axis of the sample processed at high heat input showing coarser α″ microstructure; (**e**) High magnification dark field TEM image showing nano precipitation inside α″-lath.

The average prior β-grain sizes, which ranged from 4 µm to 7 µm, were measured using a linear intercept method. In general, both intergranular and intragranular α precipitation in this transformed β-matrix were observed. At the level of magnification shown in Fig. 2, intergranular α precipitation revealed globular and lath-type morphologies. The size of globular-type α-precipitates were in the range 90 nm to 140 nm, while the width of the lath-type α-precipitates were in the range 70 nm to 100 nm, both increasing with increase in heat input. The number density of all precipitates decreased with an increase in heat input, which directly explained the trend in strength values observed. However, an in-depth investigation into the nature of the fine precipitates, as well as a study to determine whether any other types of precipitates actually give rise to such high strength, is required. Transmission electron microscopy (TEM) was performed on the samples processed under low and high heat input conditions (as shown in Fig. 3).

Unlike the SEM images (Fig. 2(b–d)), which show globular α precipitation, bright field TEM images (Fig. 3) show three different variants of lath/needle-type precipitates with lengths on the order of 230 nm, and thicknesses ranging from 30 nm to 40 nm, with extremely fine precipitation within the laths. The morphology of these precipitates led to an assumption that the microstructure contained both α-and α″-laths. Earlier work by Guo *et al*.^23^ showed the formation of α″-martensite with orthorhombic crystal structure due to high cooling rates associated with linear friction welding of Ti6246. The same α″-martensite type of microstructure was expected in this work. The orientation relationship between α″-and β-phases was given by [100]_α″_|| [100]_β_, [010]_α″_|| [1 $\bar{1}\,$0], [001]_α″_|| [110]^24–26^; and according to the crystallographic relationship between the α″-and β-phases, 12 variants of α″-phase could be expected from a parent β-phase. The dark field TEM images in Fig. 3(b–e) obtained from the <110> zone axis showed the presence of α″-martensite with two of the variants perpendicular to each other. Moreover, the number density of these laths decreased with an increase in heat input (comparison between Fig. 3(b,d)). Furthermore, high magnification dark field TEM images showed homogeneous precipitation on the order of less than a few nm. The high dislocation density in the shear deformed structure generated during FSP provided a large number of nucleation sites for precipitate formation. Li *et al*.^27^ obtained a high yield strength of 1700 MPa in a UGF beta Ti2448 alloy consisting of nanostructured α phase. The dual phase microstructure was obtained by warm rolling and a three-step heat treatment. In order to confirm the presence of these fine-scale features, high energy synchrotron radiation X-ray diffraction (SR-XRD) studies were performed on a sample processed under low heat input conditions (taken from the same location where SEM and TEM studies were conducted).

## Synchrotron Radiation X-ray Diffraction (SR-XRD) Studies

**Figure 4:**
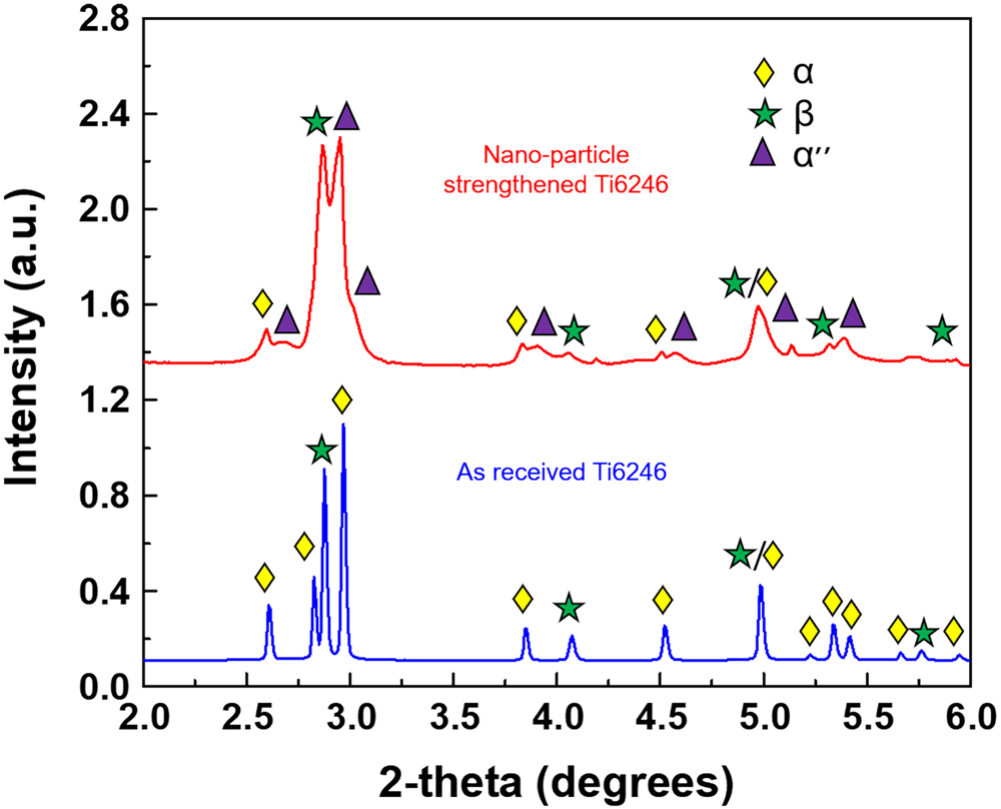
SR-XRD plots of peak intensity as a function of 2θ for the as-received (blue) and low heat input processed (red) materials.

High-energy SR-XRD studies were performed on AR sample, as well as sample processed under low heat input condition (detailed description of the instrument and experiment provided in the “Methods” section). Figure 4 shows the peak intensities as a function of 2θ for these samples along with their volume fractions. SR-XRD results confirmed that the major peaks after FSP were comprised of α″-martensite and β, which had volume fractions of approximately 67% and 22%, respectively. The volume fractions of phases are determined using Reitveld analysis (description included in the “Methods” section). SR-XRD scans also revealed significant peak broadening for FSP processed samples. Correlations between the microstructure and properties for samples processed under low heat input condition will be given emphasis on discussion section.

## Discussion

**Figure 5:**
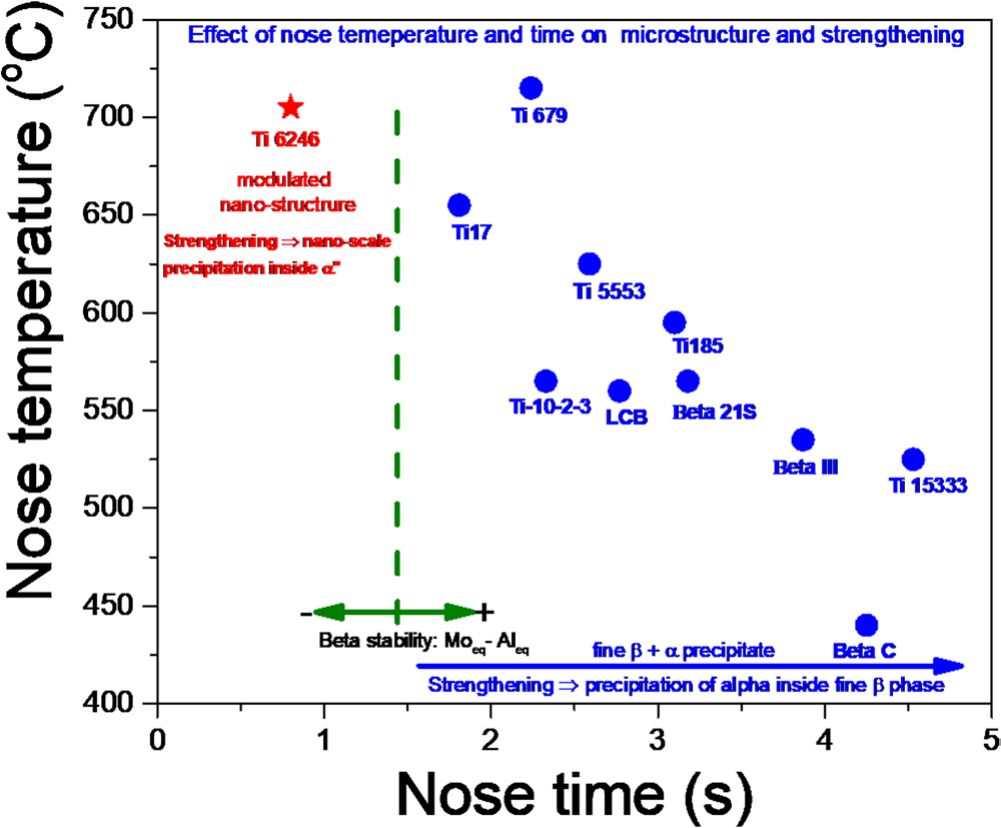
Nose temperature vs nose time plot for α precipitation in commercially available titanium alloys, indicating order for Beta stability index.

The hierarchy of properties in near-β-titanium alloys is best preserved by keeping the β-grain size (typically less than 10 µm) as fine as possible, and subsequent precipitation of α within β -grain^28^. The former was achieved by many authors through arduous thermomechanical processing^29,30^, and the latter by a two-step aging technique, which can be further enhanced by the introduction of defects (such as dislocations and stacking faults) in the prior β-grain matrix. During FSP, friction between the tool and workpiece generates extreme temperatures, which lowers the flow stress of the material and generates significant plasticity in the vicinity of the rotating tool (“Methods” section presents a detailed description of this process). This friction imparts substantial strain within the workpiece, and results in microstructural refinement. For this reason, the approach was to use FSP for refinement by quenching the high temperature β-microstructure to achieve a significant dislocation density, and subsequently performing a two-step aging treatment for uniform precipitation of α. The properties obtained after FSP were well understood in the context of their continuous cooling transformation diagrams (CCT). Based on empirical correlation by Yolton *et al*.^31^, the critical cooling rate required to quench the high temperature β-phase for many titanium alloys is shown in Fig. 5. The β phase was quenched through FSP in two of the metastable β titanium alloys; namely, Ti-185 and Beta C, (details of which are given elsewhere)^32^. In contrast, due to an excess α-stabilizing content in Ti6246, quenching the high temperature β-phase was not possible; instead, α″-martensite forms in a prior β-grain microstructure of 3 µm to 5 µm.

The microstructural evolution during FSP, which was responsible for development of α″-, β-, and α-phases in the microstructure, was observed from SR-XRD and TEM results (Fig. 3(b)). Lattice parameter of a = 3.072, b = 5.024, and c = 4.709 nm was determined for the of α″-orthorhombic martensite phase through diffraction scans. Wang *et al*.^33^ reported that when Ti2448 alloy was heated and aged in β + α phase field, the nano-scale compositional modulation resulted elastically dissimilar domains. The lattice mismatch of these domains depended on the temperature of annealing. The elastically soft solute lean phase undergoes a bcc to hcp transformation with a gradual change in the orthorhombicity of the phase as the aging time increases. Hence, the temperature and time of annealing can have a strong influence on the orthorhombicity of the phase. The degree of orthorhombicity was given by $\frac{b}{a\sqrt{3}}$, which had an average value of 0.944(compared to unity for hexagonal symmetry), with 0.948 for the specimen quenched from the high temperature β-phase field, and 0.941 for the specimen quenched from α + β phase field^26^. This indicated that the peak temperature during processing was close to (but did not exceed) the β -transus of Ti6246. The β-transus temperature for Ti6246 is 935 °C, which could be higher than the attained peak temperature. This implied that some amount of α was retained in the microstructure. However, TEM results could not identify distinct α-and α″-phases, most likely due to the low volume fraction of α-phase. For Ti6246, the martensitic start temperature is 550 °C and the cooling rate required for transformation in linear friction stir welding is 15–25 °C/s^23^. The cooling rate required to miss the nose temperature for α-phase formation is calculated as above 466 °C/s (Fig. 5), which is approximately two orders of magnitude higher than that the aforementioned value. It has been reported that cooling rates in the range of 30–40 °C/s occur in friction stir welds of titanium alloys, resulting in the formation of diffusionless transformation products^34–36^. Despite the low cooling rate in FSP, observation of the martensitic structure indicated that the resulting microstructure was a function of the extreme strain involved in the process. A sample subjected to a peak temperature close to the β-transus of Ti6246 was likely to exhibit a very high-volume fraction (66%) of α″-martensite, as in the case of the current study, and was supported by SR-XRD and TEM results. The orthorhombic α″-martensite observed had a fine acicular morphology. The dimensions (30 nm to 40 nm width) of these orthorhombic α″-martensite were believed to be the result of either small β-grains at peak processing temperatures or the presence of primary α-phases, that might hindered the growth of martensite laths^37^. FSP led to a highly complex strain resulting from deformation involving variable peak temperatures and cooling rates. This varying cooling rate portends a high possibility that this martensitic structure could be rapidly age-hardened by the precipitation of nanoscale β. In other words, auto-tempering of orthorhombic martensite during cooling was consistent with previously reported results^23,26^. For Ti-Mo alloys, it was proposed that the auto-tempering of metastable α″-structure below the M_s_ temperature involves a spinodal decomposition such that^38^,

$${\rm{\alpha }}^{\prime\prime} \to {{\rm{\alpha }}^{\prime\prime} }_{{\rm{depleted}}}+{{\rm{\alpha }}^{\prime\prime} }_{{\rm{enriched}}}\to {\rm{\alpha }}+{\rm{\beta }}$$

This suggested that the non-equilibrium conditions imposed by FSP initially created a modulated microstructure for α″, which transformed into α- and β-phases upon auto-tempering. Dark field TEM images resulted in nanoscale precipitates ranging in size from 20 to 30 nm, with an approximate interparticle spacing of less than 6 nm, which was further validated by the peak broadening observed in SR-XRD measurements^26^. This extreme peak broadening could have resulted from the large content of retained dislocation density produced during FSP^39^. The presence of nanoscale precipitates could have effectively blocked the dislocation motion, thereby increasing material strength. The outstanding mechanical properties (1996 MPa and 5% elongation) are therefore attributed to Orowan strengthening (more likely an Orowan looping mechanism due to the size scale of the precipitates), which is identified as the major precipitation hardening mechanism operational in the alloy. The modified Orowan strengthening mechanism for titanium alloys containing nanoscale precipitates can be expressed as follows^40^

1 $${\sigma }_{ps}=3.1\times 0.84\times \frac{Gb}{\delta }$$

2 $$\delta =[\frac{0.779}{\sqrt{f}}-0.785]{d}_{s}$$

3 $$\frac{{d}_{r}}{{d}_{s}}=\sqrt[3]{\frac{2}{3\lambda }}$$

where *σ*_*ps*_ is the precipitation strengthening contribution from nano-particles,

*G* is the shear modulus of precipitates, b is the burger’s vector of dislocation,

*f* is the volume fraction of nano-precipitates,

*λ* is the aspect ratio of precipitate,

*d*_*s*_ is the size of grain, and

*d*_*r*_ is the diameter of precipitate

The decrease in UTS from 1996 MPa to 1689 MPa and increase in elongation from 5–10% resulting from the increase in heat input can be explained in the following way. As the heat input was increased, the peak temperature was anticipated to have exceeded the β-transus temperature. The presence of lower number density α″ (Fig. 3) indicated that the cooling rate was not high enough to miss the nose temperature for α-phase precipitation, consequently giving rise to a mixture of α- and α″-type microstructures. Increased aspect ratios of the plates may have been attributed to the increased β grain size at the peak processing temperature. A reasonable conclusion was that fewer nanoscale precipitates formed under the medium and high heat input conditions, likely because less β stabilizing solute would be available for nano precipitation during auto-aging.

**Figure 6:**
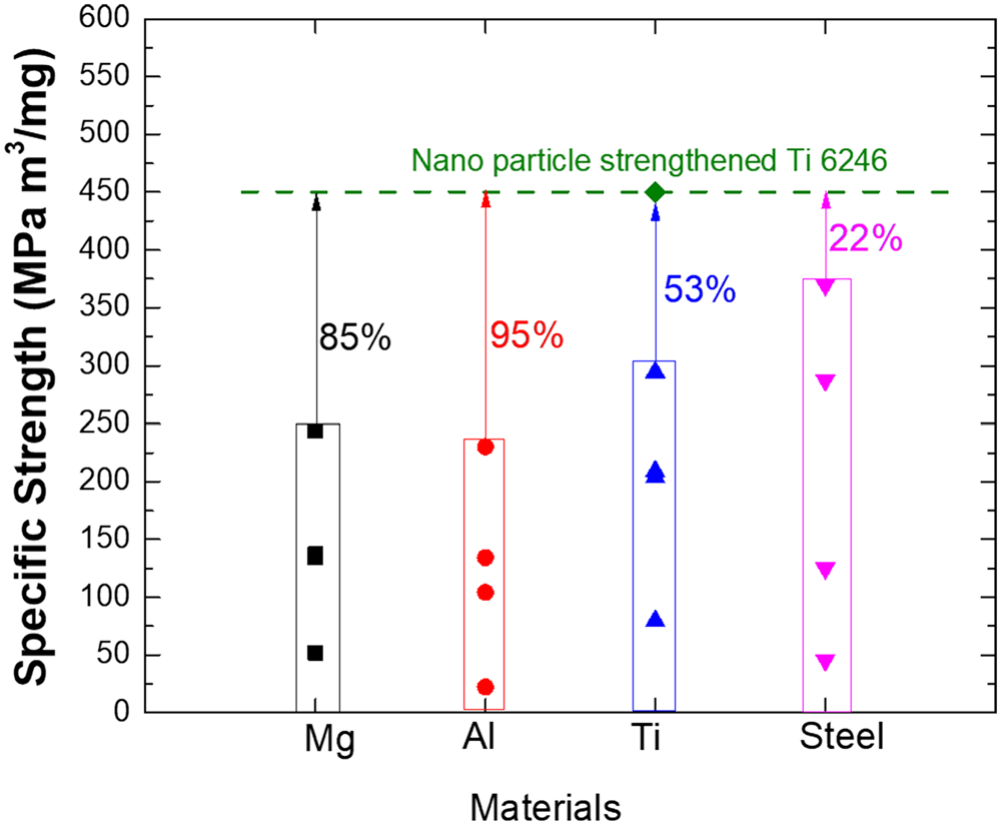
Specific strength of commercially available structural metallic material classes plotted with aid of data generated using CES software.

The highest theoretical specific strength for all metals was calculated to demonstrate the importance of the nano-particle-strengthened Ti6246 alloy in terms of its microstructure and strength. The specific strength of material is defined as the ratio of material’s strength to its density. Ti6246 achieved a significant specific strength of approximately 450 MPa m^3^/mg, which was 54% higher than any commercially available metallic materials, on average (Fig. 6). Achieve this level of specific strength would require the manufacture of a steel with an UTS of approximately 3600 MPa. This indicated that the alloy could have potential to replace structural steel for certain weight-saving applications.

To conclude, FSP was successfully performed on Ti6246, and the origin of exceptional strength was primarily due to nano-scale precipitation within α″-laths, which resulted in a modulated microstructure. This precipitation was believed to occur during auto-aging via spinodal decomposition.

## Methods

### Ti6246 material and processing

Aerospace quality Ti6246 was supplied by Arconic in the form of a billet, which was cut into several 7 mm thick pieces using wire EDM for FSP. FSP was carried out using W-1% La_2_O_3_ tool with shoulder diameter, pin diameter and pin length of 10.1 mm, 6.3 mm and 1.7 mm, respectively; at tool rotation speeds of 600 RPM, 800 RPM and 1000 RPM (which are referred to low, medium and high heat input, respectively, throughout this manuscript), and at a constant tool traverse speed of 50.8 mm/min, with a 2.5° tilt angle opposite to the processing direction. Argon gas shielding was used around the samples to prevent oxidation.

### Tensile testing

Mini-tensile samples with gauge length, width and thickness of 3 mm, 1 mm and 0.45 mm, respectively, were sectioned from the center of the SZ parallel to the processing direction. Tensile testing was performed on a custom-built mini tensile testing machine at room temperature and at an initial strain rate of 1 × 10^-3^ s^-1^.

### Microstructural characterization

Microstructural characterization of the transverse cross sections of samples was performed using FEI Nova NanoSEM 230. For TEM analysis, samples were first prepared by using the FEI Nova Nanolab 200 dual-beam focused ion beam SEM system and characterized using a FEI Titan 80–300 TEM.

Beta stability index is defined as [Mo]_*eq*_ − [Al]_*eq*_, where^7^,

4 $${[{\rm{Mo}}]}_{eq}=[{\rm{Mo}}]+\frac{2\,[{\rm{V}}]}{3}+\frac{[{\rm{Nb}}]}{3}+3\,([{\rm{Fe}}]+[{\rm{Cr}}])$$

5 $${[{\rm{Al}}]}_{eq}=[{\rm{Al}}]+\frac{[{\rm{Sn}}]}{3}+\frac{[{\rm{Zr}}]}{6}+10\,([{\rm{C}}]+[{\rm{O}}]+2\,[{\rm{N}}])$$

### Synchrotron X-ray diffraction (SR-XRD)

SR-XRD measurements were collected at the Advanced Photon Source (APS) in Argonne National Laboratory on sector 11-ID-C with a beam energy of 105 keV, (equivalent to a wavelength of 0.117418 Å) and a beam size of 0.2 × 0.2 mm^2^. Full Debye-Scherrer rings were collected using a Perkin Elmers amorphous silicon area detector positioned approximately 1.805 m from the sample over a total exposure time of 10 seconds, 1 s per frame for 100 frames, and calibrated using standard CeO_2_ powder. Reitveld refinement of the SR-XRD images was performed using MAUD software by splitting each full Debye-Scherrer diffraction pattern into 36 separate, one-dimensional spectra, 20 azimuthal degrees per spectrum; and simultaneously fitting each spectrum to the appropriate phases to produce a total representative volume fraction of the interaction volume.

### Impact-statement

Our processed alloy attained the highest specific strength of any metallic alloy system, with a value of 450 MPa-m^3^/mg.

## Data Availability

All the raw data is available with the corresponding author and can be provided on a reasonable request.
